# Genome Sequences of Three SARS-CoV-2 P.1 Strains Identified from Patients Returning from Brazil to Italy

**DOI:** 10.1128/MRA.00177-21

**Published:** 2021-03-25

**Authors:** Emiliano Delli Compagni, Lucija Jurisic, Marialuigia Caporale, Federico Bacà, Silvia Scialabba, Sara Fanì, Alessia Perullo, Michela Toro, Alice Marchegiano, Michele Martino, Daniela Di Giacomo, Teresa Romualdi, Serena Verzulli, Eugenia Ciarrocchi, Barbara Secondini, Marta Di Giuseppe, Roberta Irelli, Alessio Di Lorenzo, Fabrizia Valleriani, Shadia Berjaoui, Ilaria Puglia, Valentina Curini, Iolanda Mangone, Alessio Lorusso

**Affiliations:** a Istituto Zooprofilattico Sperimentale dell’Abruzzo e Molise, Teramo, Italy; b Faculty of Veterinary Medicine, Università degli Studi di Teramo, Teramo, Italy; DOE Joint Genome Institute

## Abstract

Severe acute respiratory syndrome coronavirus 2 (SARS-CoV-2) is the causative agent of the current coronavirus disease 2019 (COVID-19) pandemic. We report the complete sequences of three SARS-CoV-2 P.1 strains obtained from nasopharyngeal swab specimens from three patients returning from Brazil to Italy.

## ANNOUNCEMENT

Coronaviruses (CoVs) are a large group of viruses that infect many different animals. They also cause medium to severe infections in humans (1). Severe acute respiratory syndrome coronavirus 2 (SARS-CoV-2) (species *Severe acute respiratory syndrome-related coronavirus*, subgenus *Sarbecovirus*, genus *Betacoronavirus*, family *Coronaviridae*) is the causative agent of the current pandemic of CoV respiratory disease, named by the WHO coronavirus disease 2019 (COVID-19) (2, 3). SARS-CoV-2 was first identified in late 2019 in Wuhan, Hubei Province, China, and since then has spread quickly all over the globe. Since March 2020, SARS-CoV-2 variants with G614 in the S protein have replaced the D614 variants and have become the dominant form circulating globally. D614G is proposed to increase infectivity in cell assays *in vitro* and to enhance viral transmissibility in nature (4, 5). Several SARS-CoV-2 variants are now circulating globally (6), and some of them have raised international concern (7).

On 6 January 2021, the National Institute of Infectious Diseases of Japan detected a new variant of SARS-CoV-2 from four travelers who arrived in Tokyo, Japan, from Amazonas, Brazil (8). This new variant was recently revealed to be circulating in December in Amazonas state, where very high infection rates were estimated previously (9). The new lineage, named P.1 (6), contains a unique constellation of mutations, including several mutations of known biological importance, such as E484K, K417T, and N501Y, in the receptor binding domain of the S protein (10). These mutations may have an impact on the transmissibility and antigenic profile of the virus, which may affect the ability of antibodies generated through natural infection or vaccination to neutralize the virus.

Here, we describe the genome sequences of SARS-CoV-2 P.1 strains identified from three patients, belonging to the same family, who returned to the Abruzzo region from Brazil. Approval by a research ethics committee for sequencing of these strains was not required because these activities were conducted as part of the legislated mandate of the Italian Ministry of Health (protocol 0000644-08/01/2021). All procedures were carried out in accordance with the Declaration of Helsinki, as revised in 2013, for human subjects.

Swab samples (TE30939/2021, TE30964/2021, and TE30968/2021) were collected from the asymptomatic patients on 18 January 2021 and sent to the IZSAM for SARS-CoV-2 diagnosis (11). Briefly, following virus inactivation (PrimeStore molecular transport medium [MTM]; Longhorn Vaccines & Diagnostics, Bethesda, MD, USA) in a biosafety level 3 (BSL3) biocontainment laboratory, starting from a total volume of 200 μl of oropharyngeal swab transport medium (physiological solution), and nucleic acid purification (MagMax CORE kit), RNA detection was performed with the TaqMan 2019-nCoV assay kit v2 (Thermo Fisher Scientific, Waltham, MA, USA), whose results were interpreted following the manufacturer’s instructions. This test targets three different portions of the SARS-CoV-2 genome, in the open reading frame 1ab (ORF1ab) and S and N protein-encoding genes. The three samples tested positive for SARS-CoV-2 RNA, with threshold cycle (*C_T_*) values of <20 for all genome targets.

Samples were processed by next-generation sequencing by means of the Artic v3 protocol (12). Briefly, cDNA was synthesized starting from 2.25 μl of purified RNA (https://www.protocols.io/view/ncov-2019-sequencing-protocolbbmuik6w?version_warning=no). Then, cDNA was diluted 2-fold with water, and 2 μl of diluted cDNA was amplified in a 10-μl reaction mixture with Q5 high-fidelity DNA polymerase (NEB) using each of two Artic v3 primer pools tiling the SARS-CoV-2 genome. Library preparation was carried out by using the DNA prep (M) tagmentation kit (24 samples) (Illumina, Inc., San Diego, CA, USA). Deep sequencing was performed on the MiniSeq system (Illumina, Inc.) with the MiniSeq midoutput kit (300 cycles) and standard 150-bp paired-end reads. For bioinformatic analyses, all tools were run with default parameters unless otherwise specified. Quality control of the reads was performed using FastQC. Reads obtained were trimmed by Trimmomatic (13). SARS-CoV-2 consensus sequences were obtained using iVar (14) after reads were mapped to the reference sequence (Wuhan-Hu-1 [GenBank accession number NC_045512]) by Snippy (15). Total numbers of raw reads ranged from 996,070 to 1,069,256, with an average base quality score of 36.65. The numbers of mapped reads (151 nucleotides [nt] in length) ranged from 316,377 to 369,432, with coverage depth ranging from 3,529× to 4,149×. Overall, consensus sequences were complete for the whole SARS-CoV-2 genome. Only in the TE30964/2021 consensus sequence, a 247-nt stretch of Ns was present in the ORF1b region at positions 16496 to 16743. By MegAlign Pro (Laseregene; DNASTAR, Madison, WI, USA), the consensus sequences showed 100% nucleotide identity to each other across the whole genome. Sequences were submitted to the Pangolin COVID-19 lineage assigner (https://pangolin.cog-uk.io) and assigned to the P.1 lineage. Sequences were released on 26 January 2021 to the GISAID database and later to the NCBI database. The amino acid mutations with respect to the reference sequence are reported in Table 1.

**TABLE 1:** Mutations of strains TE30939/2021, TE30964/2021, and TE30968/2021, compared with the Wuhan-Hu-1 SARS-CoV-2 reference sequence (GenBank accession number NC_045512)

Protein	Mutation(s)
Spike	D138Y, D614G, E484K, H655Y, K417T, L18F, N501Y, P26S, R190S, T20N, T1027I, V1176F
N	G204R, P80R, R203K
NS3	S253P
NS8	E92K
NSP3	K977Q, S370L, T133I
NSP6	F108del, G107del, S106del
NSP12	P323L
NSP13	E341D

Here, we describe the SARS-CoV-2 P.1 strains obtained from travelers returning from Brazil. Sequences were released a few days after the collection of samples. We strongly believe that timely sharing is essential for tackling the global pandemic in light of the role that specific mutations may have in the pathobiology of the virus.

### Data availability.

The whole-genome sequences were deposited in the GISAID database under the accession numbers EPI_ISL_875568 (hCoV-19/Italy/ABR-IZSGC-TE30968/2021), EPI_ISL_875567 (hCoV-19/Italy/ABR-IZSGC-TE30964/2021), and EPI_ISL_875566 (hCoV-19/Italy/ABR-IZSGC-TE30939/2021). The sequences were deposited in the NCBI database under the accession numbers MW642248 (TE30939/2021), MW642249 (TE30964/2021), and MW642250 (TE30968/2021). The SRA accession number is PRJNA704235.
